# Faintings and Fits

**Published:** 1887-03-19

**Authors:** 


### TILL THE DOCTOR COMES.

[Inquiries and Communications for this column should be addressed, "Physician," care of the Editor of THE HOSPITAL.]

### XIII.—FAINTINGS AND FITS.

*Fainting Attacks.*—Caused generally by an insufficient supply of blood to the brain. Keep the head very low—lower than the body. Give hot brandy and water; put smelling salts, etc., to the nose, and slap the forehead with a cold wet towel, or dash cold water on the face. Open the windows wide. Rub the limbs from below upwards to send the blood to the heart and brain. Give stimulants as soon as the patient can swallow.

*Epileptic Fits.*—These vary very much in degree, and may consist only of loss of consciousness for a few moments, when the patient recovers as suddenly as he became ill, but remembers nothing of the interval. This requires no treatment, except seeing that the patient does not hurt himself. But in what is generally known as an epileptic fit, the patient falls, foams at the mouth, struggles a great deal, becomes blue in the face, and when the fit is over he remains in a dazed, stupid state for some time. There is too great a tendency to struggle with such patients, to hold them down and keep them quiet. This is a great mistake; it does far more harm than good. The patient should, if possible, be placed in the middle of a large bed, and care taken that he does not hurt himself in his struggles, particularly that he does not strike his arms violently against the sides of the bed. If, however, he falls when out of doors, roll up a coat, or put something soft under his head, and control the limbs sufficiently to prevent him from injuring himself, but no more. Put a cork or something between his teeth to prevent him from biting his tongue. Do not attempt to make him swallow anything. Unfasten his clothes, especially about the neck and chest. Persons subject to these fits should not be employed in any work in which they are likely to injure themselves on the sudden advent of a fit.

*Hysterical Fits.*—Almost always occur in young girls, and can generally be distinguished from epileptic fits by opening the lids, and touching the ball of the eye. In hysteria this part is sensitive, and the patient will wince, but in epilepsy all sensation is abolished, and the patient will not feel anything at all. Hysterical people, also, never bite their tongue nor hurt themselves. If, however, there is any doubt, it will be safer to treat the case as an epileptic fit. In true hysteria a few sharp strokes across the face with a cold wet towel, or some cold water poured from a height on to the face, will usually cut short the fit, if you speak firmly to the patient at the same time. Do not treat such patients harshly, but be very firm with them, and above all, never commiserate them; this will only make them a great deal worse. As long as sympathetic but unwise friends will rub their limbs, kiss them, and condole with them, so long will the fit last.

